# Evaluation of the diagnostic efficiency of voided urine fluorescence *in situ* hybridization for predicting the pathology of preoperative “low-risk” upper tract urothelial carcinoma

**DOI:** 10.3389/fonc.2023.1225428

**Published:** 2023-07-27

**Authors:** Ben Xu, Jia-En Zhang, Lin Ye, Chang-Wei Yuan

**Affiliations:** Department of Urology, Peking University First Hospital, Beijing, China

**Keywords:** fluorescence *in situ* hybridization, upper tract urothelial carcinoma, tumor stage, tumor grade, low-risk

## Abstract

**Objectives:**

To evaluate the clinical utility of voided urine fluorescence *in situ* hybridization (FISH) for predicting the pathology of preoperative “low-risk” upper tract urothelial carcinoma (UTUC).

**Methods:**

Information of patients preoperatively diagnosed with “low-risk” UTUC receiving radical nephroureterectomy (RNU) between May 2014 and October 2019 were retrospectively collected. All of the patients accepted the FISH test and then were divided into two groups according to the results of FISH. The diagnostic value of FISH was assessed through the receiver operating characteristics (ROC) curve and area under the curve. Logistic regression analysis was applied to examine FISH as a predictive factor of tumor final stage and grade of preoperative “low-risk” UTUC.

**Results:**

In total, 129 patients were included. Of them, 70 (54.2%) were marked with positive FISH result. The difference at final pathology in tumor stage and tumor grade between these two groups of FISH (-) and FISH (+) had significantly statistical significance (p<0.001). Regarding to the tumor stage at final pathology, the sensitivity, specificity, positive predictive value and negative predictive value of FISH were 70.7 (58.9-80.3)68.5 (54.3-80.1)75.7 (63.7-84.8) and 62.7 (49.1-74.7), respectively. Regarding to the tumor grade at final pathology, the sensitivity, specificity, positive predictive value and negative predictive value of FISH were 64.7 (53.5-74.6), 65.9 (50.0-79.1), 78.6 (66.8-87.1) and 49.1 (36.5-62.3), respectively. The results of logistic regression analysis indicated that FISH could predict the pathologic characteristics of preoperative “low-risk” UTUC independently.

**Conclusions:**

FISH was qualified with relatively high diagnostic estimates for predicting tumor stage and grade of preoperative “low-risk” UTUC, and could be an independent predictive factor in clinical practice. For preoperative “low-risk” UTUC patients but with positive FISH result, choosing nephron-sparing surgery may require special caution.

## Introduction

Upper tract urothelial carcinoma (UTUC) is a rare tumor in the urogenital system, and its incidence rate accounts for 5% - 10% of all urothelium cancers (1). The standard surgical method is radical nephroureterectomy (RNU), and in the recently published AUA guidelines (2), clinicians should give neoadjuvant cisplatin based chemotherapy or adjuvant cisplatin based chemotherapy. At present, the tumor specific survival rate for late stage or high grade is still low, and the tumor specific survival rate for pathological T2/T3 UTUC is less than 50% (3).

Due to the fact that RNU can lead to renal dysfunction in patients (4), there has been controversy in recent years over whether RNU is necessary for UTUC. For tumors with a single, small diameter, and no clear imaging invasive evidence, choosing nephron-sparing surgery can not only avoid the complications caused by RNU, but also exhibit no clear difference in the 5-year tumor specific survival rate compared to RNU (5). Therefore, there is currently a tendency to classify preoperative UTUC patients into “high-risk” and “low-risk” according to preoperative relevant factors to guide precise treatment strategies (6). In the current EUA and the latest AUA/SUO guidelines (2), preoperative “low-risk” patients need to simultaneously meet the requirements of (1) a single tumor, (2) no high-grade tumor detected by cytology, and (3) no tumor infiltrative growth detected by CT examination. However, for preoperative “low-risk” UTUC patients using nephron-sparing surgery, postoperative pathology may indicate totally opposite pathological results– high stage or high grade, and thus the postoperative recurrence rate of such patients is significantly increased (7). Therefore, the existing criteria for judging “low-risk” UTUC preoperatively may still be insufficient, and other new methods urgently need to be applied to minimize the occurrence of preoperative “low-risk” with postoperative “high-risk” situations.

Fluorescence *in situ* hybridization (FISH) of urine exfoliated cells is one of the important diagnostic methods of UTUC. Studies have shown that FISH has a higher diagnostic ability in UTUC than lower urothelial cancer, and is superior to urine exfoliated cells in predicting high-risk UTUC (8, 9). In the year of 2017, Su XH et al. from our centers were the first throughout the world to report the use of FISH to predict the pathological stage and grade of UTUC after surgery (10). And in the year of 2018, Guan B et al. considered that the urinary FISH test could be a powerful tool in predicting the risk of bladder recurrence and the prognosis in patients with UTUC (11). Nevertheless, the biggest flaw of the article lies in its limited guiding significance for practical clinical work. The patients included in the above research were consist of preoperative “low-risk” and “high-risk” groups, especially “high-risk” group. Based on the fact that these “high-risk” patients themselves were originally arranged to undergo RNU, so whether preoperative FISH test is positive or not is not of great significance for guiding clinical work. To surprise, this study will focus on exploring the predictive effect of FISH technology on postoperative pathological conditions only in preoperative “low-risk” UTUC populations, which will greatly help doctors choose surgical methods more accurately. On the one hand, it can avoid excessive RNU, and on the other hand, it can also help reduce the risk of high postoperative recurrence in patients who undergo nephron-sparing surgery by a precise division. So far, this is the first article worldwide to use FISH testing to predict postoperative pathological stage and grade for preoperative “low-risk” UTUC patients. The relevant conclusions may use the FISH test results as a new tool for accurately dividing preoperative patients into “low-risk” or “high-risk” group in the future, which may play a significant impact on the traditional standards of preoperatively clinical risk stratification.

## Materials and methods

This study retrospectively collected the data of patients who were preoperatively diagnosed with “low-risk” UTUC and underwent RNU in our center from May 2014 to October 2019. All of the patients underwent voided urine FISH and urine cytology within one week before surgery. Inclusion criteria: (1) The surgical method was RNU; (2) The postoperative pathology was urothelial carcinoma; (3) The FISH inspection results were clear and complete; (4) Preoperative imaging examination simultaneously met the requirements of single tumor, no hydronephrosis, and no clear infiltrative growth in computer tomography; (5) No high-grade tumors were detected in preoperative urine exfoliative cytology examination. Exclusion criteria: (1) Those whose pathological results cannot determine the tumor grade and depth of tumor infiltration; (2) Those who only underwent ureteroscopy to obtain biopsy pathological results. This study had been approved by the Ethics Committee of our center (Peking University First Hospital) (No.2023-182-001). Patient consent was not required by the ethics committee due to the retrospective nature of the study.

**Figure 1:**
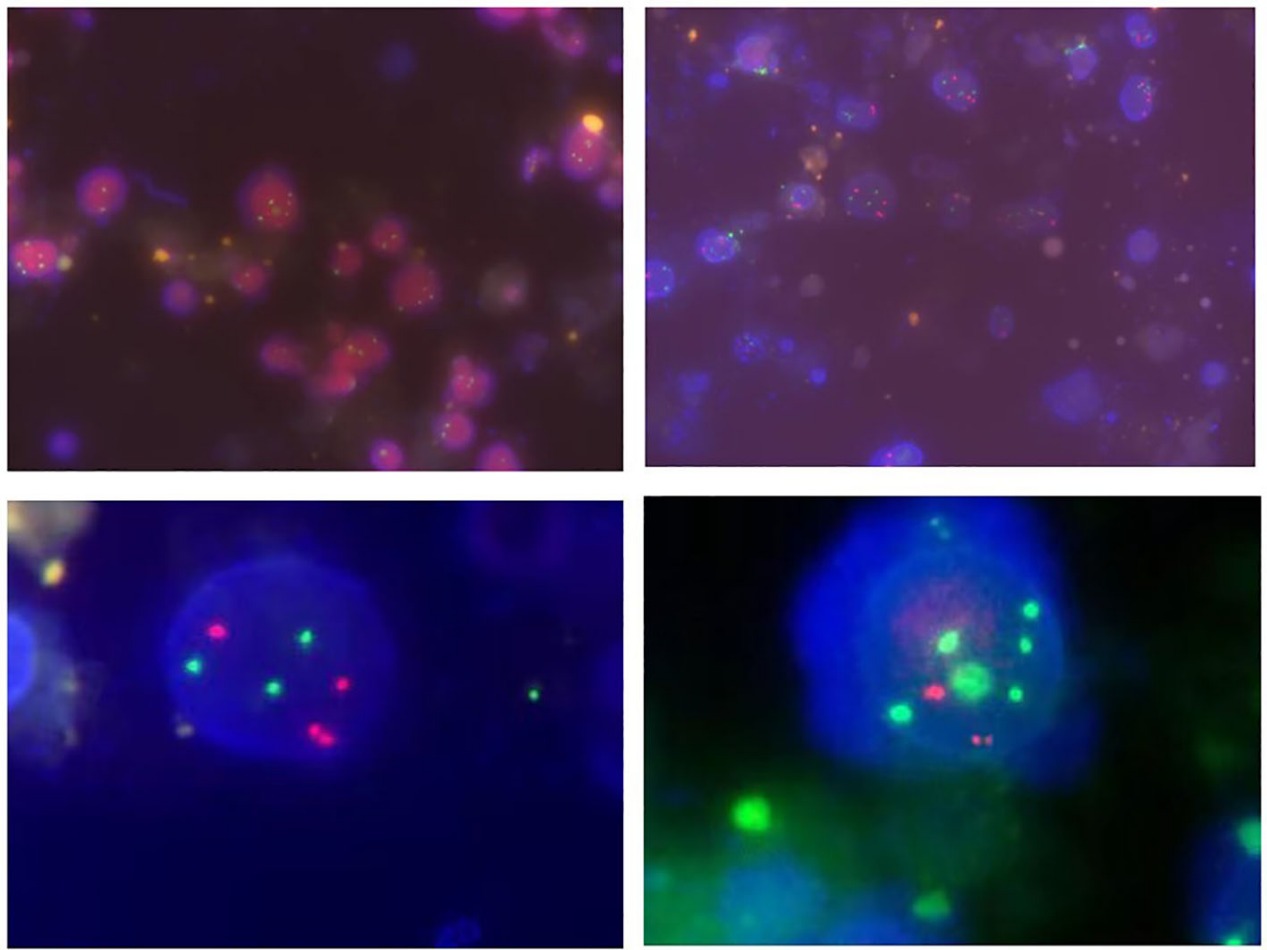
Representative diagram of FISH (+) patients.

Approximately 50-200ml of initial morning urine were collected from patients, and the FISH testing process was performed using a commercial UroVysion kit produced by Vysis in the United States. The appearance of cells with abnormal morphology under fluorescence microscopy was characterized by large nuclear volume, abnormal morphology, uneven DAPI staining, and cell clusters. Such cells were searched under a microscope and at least 25 cells were analyzed with abnormal morphology. The signal distribution of cells with abnormal morphology was manifested as three or more signals from one or more probes, while chromosomal increase refers to the specific colors displayed by various probes under fluorescence microscopy (such as red on chromosome 3, green on chromosome 7, blue or green on chromosome 17), or homologous deletions at the 9p21 locus. The analysis continued until 4 cells were detected to have multiple chromosomal additions or 12 cells had 9p21 homologous deletions, or the entire sample was analyzed. The corresponding results were recorded as positive or negative (12). The representative diagram of positive patients was shown in **Figure 1**.

The counting data were described in the form of quantity and percentage, and the patients’ age was converted into two categorical variable with 65 years as the critical value. The differences in clinical pathological information between the FISH positive and negative groups were compared using Pearson’s chi square test or Fisher’s exact test. The receiver operating characteristic (ROC) curve and area under curve (AUC) were used to evaluate the diagnostic efficacy of FISH test in determining tumor stage and grade. Other diagnostic indicators included sensitivity, specificity, positive predictive value, and negative predictive value. Multivariate logistic regression analysis clarified whether FISH test results could serve as an independent predictor of tumor stage and grade. All data were analyzed using SPSS 22.0 software, with a statistically significant difference of P<0.05.

## Results

A total of 129 patients with UTUC were enrolled in this study, including 80 (62.0%) males and 49 (38.0%) females, ranging in ages from 41 to 86 years old. The number of FISH positive patients was 70, accounting for 54.2% of the total. The study subjects were divided into two groups based on positive and negative FISH results. Among the FISH positive group, 61 (87.1%) had initial symptoms, including 57 (57/61, 93.4%) gross hematuria and 4 (4/61, 6.6%) back pain. The corresponding results for the FISH negative group were 48 (81.4%), all of whom were consulted with the gross hematuria. The surgical pathological results showed that there were 53 (75.7%) cases with stage ≥ T2 in the FISH positive group, 55 (78.6%) cases with high-grade tumors, while the corresponding data for the FISH negative group were 22 (37.3%) cases and 30 (50.8%) cases. Among the detailed information of variant histologies (VH) based on the novel WHO 2022, 1 case of squamous differentiation (1/30, 3.3%) was detected in the FISH negative group. Besides, 2 case of squamous differentiation (2/55, 3.6%), 1 case of adenoid differentiation (1/55, 1.8%) and 1 case of sarcomatoid differentiation (1/55, 1.8%) were detected in the FISH positive group. Above all, the difference at final pathology in tumor stage and grade between these two groups of FISH (-) and FISH (+) had significant statistical significance (p<0.001). The distribution of clinical pathological information between the two groups of patients was shown in **Table 1**.

**Table 1:** The baseline characteristics of Fish(+) and Fish (-) patients.

Characteristics		FISH(-)	FISH(+)	*P value*
Age	≥65	32 (54.2%)	32 (45.7%)	0.380
	<65	27 (45.8%)	38 (54.3%)	
Gender	Male	39 (66.1%)	41 (58.6%)	0.335
	Female	20 (33.9%)	29 (41.4%)	
Symptoms	(+)	48 (81.4%)	61 (87.1%)	0.366
	gross hematuria	48(100%)	57(93.4%)	
	back pain	0	4(6.6%)	
	(-)	11 (18.6%)	9 (12.9%)	
Tumor Location	pelvic	37 (55.9%)	47 (61.4%)	0.816
	ureter	22 (32.2%)	23 (28.6%)	
Tumor stage	≥T2	22 (37.3%)	53 (75.7%)	<0.001
	<T2	37 (62.7%)	17 (24.3%)	
Tumor grade	high	30 (50.8%)	55 (78.6%)	0.001
	variant histology			
	squamous	1(3.3%)	2(3.6%)	
	adenoid	0	1(1.8%)	
	sarcomatoid	0	1(1.8%)	
	other	0	0	
	low	29 (49.2%)	15 (21.4%)	

**Figure 2:**
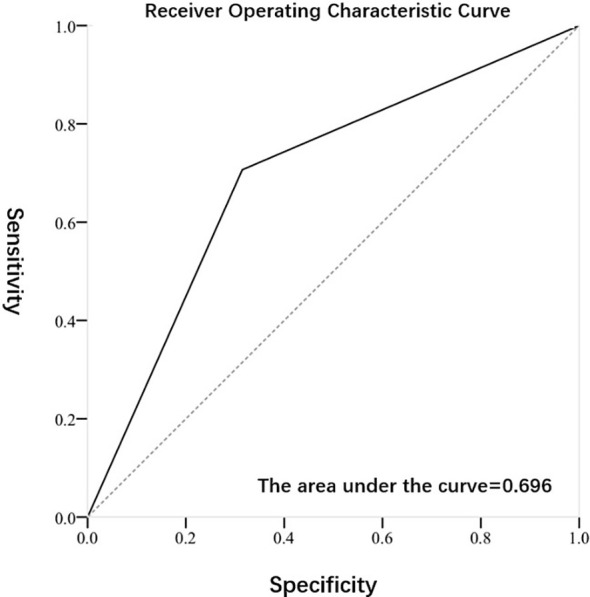
Receiver Operating Characteristic Curve of subjects predicted by FISH for tumor stage.

**Figure 3:**
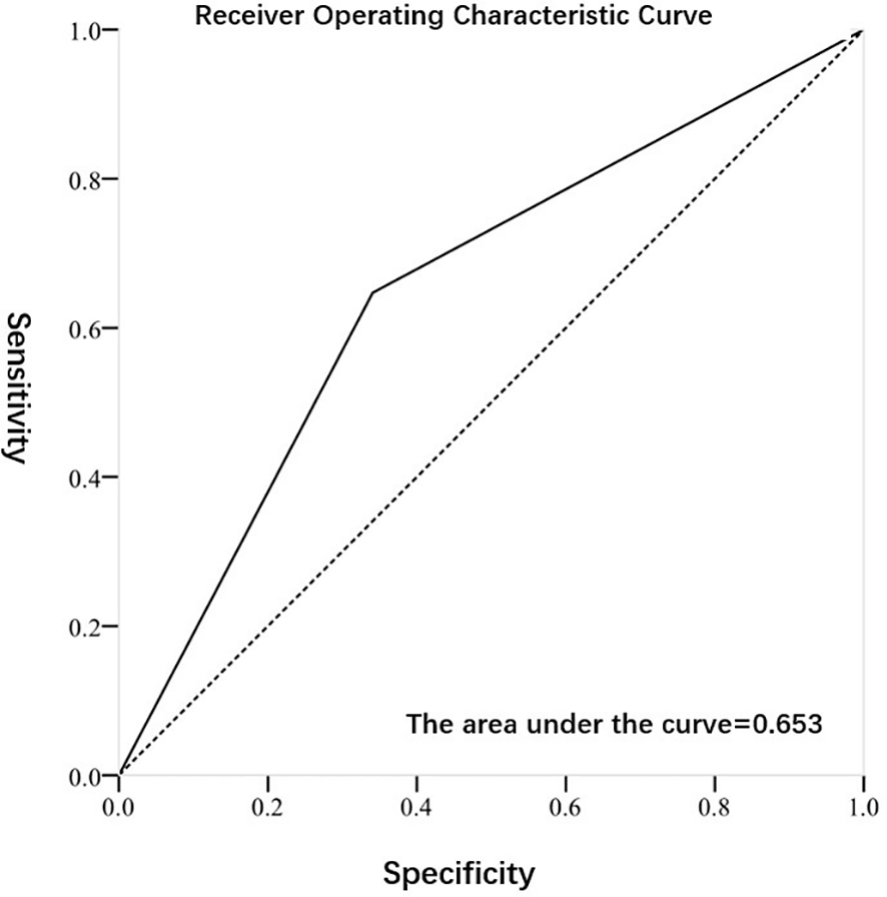
Receiver Operating Characteristic Curve of subjects predicted by FISH for tumor grade.

**Figures 2**, **3** exhibited the ROC curves of tumor stage and grade based on FISH results. The AUC value for distinguishing tumor stage ≥ T2 and<T2 was 0.696, while the AUC value for distinguishing tumor grade high and low was 0.653. **Table 2** summarized the diagnostic efficacy of FISH in determining tumor stage and grade at final pathology. Regarding to the tumor stage at final pathology, the sensitivity, specificity, positive predictive value and negative predictive value of FISH were 70.7 (58.9-80.3), 68.5 (54.3-80.1),75.7 (63.7-84.8) and 62.7 (49.1-74.7), respectively. Regarding to the tumor grade at final pathology, the sensitivity, specificity, positive predictive value and negative predictive value of FISH were 64.7 (53.5-74.6), 65.9 (50.0-79.1), 78.6 (66.8-87.1) and 49.1 (36.5-62.3).

**Table 2:** Diagnostic indicators for predicting tumor stage and grade using FISH.

	Sensitivity	Specificity	Positive predictive value	Negative predictive value
Stage	70.7 (58.9-80.3)	68.5 (54.3-80.1)	75.7 (63.7-84.8)	62.7 (49.1-74.7)
Grade	64.7 (53.5-74.6)	65.9 (50.0-79.1)	78.6 (66.8-87.1)	49.1 (36.5-62.3)

**Table 3** exhibited the results of univariate and multivariate logistic regression analysis, with age, gender, initial symptoms and tumor location as confounding factors in the multivariate analysis. This analysis indicated that FISH could serve as an independent factor in predicting tumor stage [OR value: 5.312 (95% confidence interval: 2.135-13.214), P-value:<0.001] and grade (OR value: [2.813 (95% confidence interval: 1.219-6.491), P-value: 0.015].

**Table 3:** Logistic regression analysis of FISH for predicting tumor stage and grade.

	Univariate analysis		Multivariate analysis	
	Ratio (95% confidence interval)	*P* value	Ratio (95% confidence interval)	*P* value
Stage	5.243 (2.453-11.206)	<0.001	5.312 (2.135-13.214)	<0.001
Grade	3.544 (1.648-7.623)	0.001	2.813 (1.219-6.491)	0.015

## Discussion

“High-risk” UTUC is characterized by high tumor stage and grade, and may be accompanied by features such as tumor size exceeding 3cm, wide basal area, and concomitant carcinoma in situ, which has a relatively higher recurrence rate and mortality rate (13). “Low-risk” UTUC is positioned as a single low-grade tumor without evidence of deep infiltration. Conservative treatment methods can be adopted to preoperative “low-risk” patients, such as ureteroscopic renal pelvis tumor cauterization, local ureterectomy and anastomosis (14, 15). Therefore, the preoperative comprehensive evaluation of UTUC is crucial for the selection of subsequent treatment. This study analyzed the predictive ability of FISH for the postoperatively pathological tumor stage and grade of preoperative “low-risk” UTUC. The results indicated that preoperative FISH as an independent risk factor had a high sensitivity and specificity in distinguishing postoperative tumor stage and grade tumors in UTUC.

In the diagnosis of UTUC, FISH and urinary cytology are both commonly used to detect cancer cells (16). Previous reports have shown that FISH is more sensitive than urine cytology in diagnosing UTUC, but their specificity is similar (17). At present, FISH has become the main role in the diagnostic process of UTUC (12, 18), which can clarify the presence of cancer cells in the urinary system and qualitatively analyze space occupying lesions in combination with imaging examinations. However, there are relatively few studies on FISH to evaluate the pathological characteristics of UTUC. A retrospective study by Johannes et al. showed that the sensitivity of residual urine FISH results in predicting low-grade UTUC was 60.0%, while the sensitivity in predicting high-grade UTUC was 50.0% (19). However, in Chen et al.’s study, the sensitivity of FISH results in predicting high-grade UTUC was as high as 79.0%, higher than the 41.0% of low-grade UTUC (20). A study of 212 patients included by Eismann L et al. in 2021 showed that FISH had an important predictive role in postoperative pathological staging of UTUC (21). Above all, these studies demonstrated that preoperative FISH results have a good diagnostic ability for the pathological characteristics of UTUC.

In the reported studies, the FISH positive rate ranged from 35% to 87.5% (22–24), and the FISH positive rate in this study was 54.3%, consistent with the previous research results. In addition, the results of this study showed that the positive predictive values of FISH for predicting tumor stage and grade were both higher than 70%. It indicated that for FISH positive patients, the likelihood of the final pathological being a “high-risk” tumor was higher. Therefore, for such patients, it may not be appropriate to choose nephron-sparing surgery although these patients were classified into “low-risk” group based on the traditional risk stratification methods. Instead, it is more recommended to use RNU to avoid a significant increase in the risk of tumor recurrence due to the high stage and grade of the tumor after local resection. However, the negative predictive value of FISH in tumor stage and grade is relatively low, so although in patients with negative FISH results, there is still a certain proportion of patients who end up with “high-risk” UTUC. Therefore, in the clinical practice, it cannot easily make conservative treatment decisions only based on FISH negative cases. This study further through regression analysis denoted that FISH could serve as an independent factor in predicting tumor stage and grade. Of course, a single clinical indicator cannot achieve the accuracy of predicting pathological features. Therefore, in the future, FISH should be combined with other clinical indicators to form a prediction model to improve accuracy.

Given that the pathological stage and grade of tumors are recognized as the most critical prognostic factors for UTUC (25–30), any factors that might potentially have an impact on tumor stage and grade must be fully considered. Therefore, based on the conclusion drawn from this study “FISH results can predict the postoperatively pathological stage and grade of tumors in preoperative “low-risk” UTUC patients”, nephron-sparing surgery should be chosen with great caution even if the patient was considered as traditional “low-risk” before surgery but with FISH positive test. In the future, incorporating the results of FISH testing into the differentiation criteria of “low-risk” and “high-risk” may further reduce the risk of increased tumor recurrence due to nephron-sparing surgery.

What’s more, VH is a driver of progression and biological aggressiveness in urothelial carcinoma including UTUC and bladder cancer. UTUC with VH is rare but has been increasingly shown to confer worse prognoses, and standardized approaches to treatment for UTUC with VH have not been established. Eric Song et al. (31) has proposed that patients with VH were more likely to present at advanced stages and experience higher mortality rates when compared to pure UTUC. Antoin Douglawi et al. (32) also denoted that VH can be found in 10% of patients with UTUC and is an independent risk factor for metastasis following RNU. However, the overall survival rates and the risk of urothelial recurrence in the bladder or contralateral kidney were not affected by the presence of VH. Two recent studies (33, 34) have evaluated the different prognostic impact of VH for non muscle-invasive and muscle-invasive bladder cancer, and found that BCG treatment could be proposed considering the need for more intensive oncological surveillance when dealing with the non muscle-invasive with VH. However, clearly associated with features of more aggressive behavior, VH had not any significant impact in survival expectancies after radical cystectomy. Francesco Claps insisted that (35) more than 25% of patients harboured a VH at time of radical cystectomy. Compared to pure urothelial carcinoma, clear-cell, plasmacytoid, small-cell and sarcomatoid VHs were associated with worse disease-free survival, while lymphoepithelioma-like VH was characterized by a disease-free survival benefit. Accurate pathological diagnosis of VHs may ensure tailored counselling to identify patients who require more intensive management. Currently, in the VH assessment of UTUC, due to the rare proportion, FISH detection is not yet available to predict the rate of VH. In this trail, due to the limited number of UTUC with VH, only 5 out of 129 patients were unable to draw meaningful statistical conclusions. However, we can see a tendency that FISH positive patients are more likely to develop UTUC with VH. Given that other studies (31–35) have denoted that more active treatment is needed in urothelial carcinoma with VH, we need to be more cautious when adopting nephron-sparing surgery for low-risk UTUC with FISH (+), avoiding delaying treatment for some UTUC patients with potentially concomitant VH, although this conclusion cannot be solidly confirmed due to the small sample size of UTUC with VH at present.

The drawback of this study exhibits that it is a retrospective study with inevitable bias only based on a single center setting. At the same time, the relatively lower sample size also affects the accuracy of the data to a certain extent. Admittedly, low sample size, single center setting and its retrospective nature hindered the actual value of this research. In addition, not all of the patients with suspected UTUC in clinical practice have undergone FISH testing, thus causing partial bias in patient selection. It is still unknown whether implementing nephron-sparing surgery can truly benefit patients with negative preoperative FISH. Nevertheless, this study preliminarily confirmed the high accuracy of FISH as a preoperative examination for predicting the pathological tumor stage and grade of preoperative “low-risk” UTUC, and can serve as an independent predictor. Further large-scale and prospective studies with more center settings are urgently needed to further clarify the role of FISH examination.

## Conclusions

FISH was qualified with relatively high diagnostic estimates for predicting stage and grade of preoperative “low-risk” UTUC, and could be an independent predictive factor in clinical practice. For preoperative “low-risk” patients but with positive FISH result, choosing nephron-sparing surgery may require special caution.

## Data availability statement

The raw data supporting the conclusions of this article will be made available by the authors, without undue reservation.

## Ethics statement

This experiment was approved by the Ethics Committee of our center (Peking University First Hospital) (No.2023-182-001). Patient consent was not required by the ethics committee due to the retrospective nature of the study.

## Author contributions

BX carried out the design of this research, analysis and interpretation of data, and drafted the manuscript. J-EZ participated in the collection of data and data analysis. LY and C-WY assisted in the design of this research and project development. All authors read and approved the final manuscript.
